# Drainage and Dressing of Wounds in Minor Surgery

**Published:** 1910-09

**Authors:** 


**Drainage and Dressing of Wounds in Minor Surgery**

Archibald E. Isaccs, New York, *Medical Record,* July 16, 1910, thinks that the principles of surgery with reference to dressings and drainage are often overlooked. Drainage is assistance given to nature in providing and keeping up an unobstructed outlet for the exudate from any wound. Discharge allowed to stagnate or collect under tension would do harm. Packing is not generally required. It is only useful to keep open collapsible cavities. Dressings are applied to suppurative wounds to protect the wound from external influences, and to provide means of absorption and removal of exudate from the wound. Methods of drainage should be such as aid nature. Many wounds would do better undrained and unpacked. Gauze is open to the objection that it easily becomes stuck to the wound, and the discharge dries in its meshes and prevents rather than aids drainage. It makes a good drainage material only when it is covered with a wet dressing. . . . . A cigarette drain is better than plain gauze, because it does not adhere, and the fluid finds its way out around the gutta percha tissue. It does not give pain or do damage on its withdrawal. Gutta percha tissue is an ideal drainage material when a packing is not required. It keeps the wound open and prevents adhesion to the raw surfaces. Its smooth surface permits of easy introduction and removal. . . . In burns gutta percha tissue makes the best of dressings, because it does not adhere and allows of drainage of the fluids.

